# Recent advances of precision immunotherapy in sepsis

**DOI:** 10.1093/burnst/tkaf001

**Published:** 2025-01-13

**Authors:** Antonios Arapis, Dimitrios Panagiotopoulos, Evangelos J Giamarellos-Bourboulis

**Affiliations:** 4th Department of Internal Medicine, National and Kapodistrian University of Athens, Medical School, ATTIKON University General Hospital, 1 Rimini Str/124 62, Athens, Greece; 3rd Department of Obstetrics and Gynecology, National and Kapodistrian University of Athens, Medical School, ATTIKON University General Hospital, 1 Rimini Str/124 62, Athens, Greece; 4th Department of Internal Medicine, National and Kapodistrian University of Athens, Medical School, ATTIKON University General Hospital, 1 Rimini Str/124 62, Athens, Greece; Hellenic Institute for the Study of Sepsis, 17 Laodikeias Str/115 28 Athens, Athens, Greece

**Keywords:** Precision immunotherapy, Sepsis, Anakinra, Nangibotide, Nivolumab, Interferon-gamma, Growth factors, Adrecizumab

## Abstract

Precision immunotherapy signifies the administration of the required type of immune intervention tailored to the state of immune activation at the appropriate time window. The classification of patients into the different states of immune activation is usually done by either a protein blood biomarker or a molecular blood endotype that is diagnostic of the precise immune state. Evidence coming from trials of the last decade suggests that immune interventions should be split into strategies aiming to attenuate the exaggerated immune responses, restore sepsis-induced immunoparalysis (SII) and restore the vascular tone. Suggested strategies to attenuate the immune responses are anakinra, nangibotide and tocilizumab. Biomarkers that guide their use are ferritin, soluble triggering receptor expressed on myeloid cells-1 and C-reactive protein. Suggested strategies to restore SII are nivolumab, recombinant human interferon-gamma, CYT107, granulocyte macrophage colony stimulating factor and IgM-enriched immunoglobulin prepapations. Biomarkers that guide their use are the expression of the human leukocyte antigen DR on blood monocytes, the absolute lymphocyte count and blood levels of immunoglobulin M. One recently suggested strategy to restore vascular tone is adrecizumab, the use of which is guided by blood levels of bio-adrenomedulin. The use of these precision treatment strategies is still hampered by the need for large-scale randomized controlled trials.

Highlights

- Precision immunotherapy in sepsis requires the proper recognition of the immune state.
- Patients are often classified to predominantly pro-inflammatory sepsis and sepsis-induced immnoparalysis.
- Ferritin and soluble triggering receptor expressed on myeloid cells-1 are used for the recognition of excess pro-inflammation.
- The absolute lymphocyte count, human leukocyte antigen-DR and immunoglobulin M are used for the recognition of immunoparalysis.
- Large-scale phase 3 trials are warranted.

## Background

The strategy of immunotherapy for the management of sepsis was introduced almost 30 years ago. The concept was developed on the background of successful animal experiments showing that pre-treatment with drugs that block components of the inflammatory cascade led to survival benefit. The results of randomized controlled trials (RCTs) were not as satisfactory as expected. Agents blocking cytokines, like anti-tumor necrosis factor (TNF) antibodies, soluble TNF receptors and anakinra failed to demonstrate survival benefit [1]. Treatment with recombinant human activated protein C, namely drotrecogin-alpha, showed 6.1% absolute survival benefit. These results led to the approval of its use [2]. However, the failure to repeat the drug benefit in subsequent trials ended with the retraction of the drug from the market [3].

Does this mean that immunotherapy is a strategy that does not work in sepsis? The results of RCTs should be interpreted with caution and taking into consideration the characteristics of the studied patient population. In most RCTs no stratification of study participants into sepsis phenotype or endotype applies. Perhaps, the correct interpretation of the failing RCTs of the past is that the studied drugs do not have universal application for all patients [4]. This means that subgroups of patients experiencing a similar mechanism of immune activation may receive benefit from a given treatment. This approach is called precision immunotherapy. Some evidence that a precision approach tailored to specific needs may be beneficial was built during the COVID-19 pandemic.

The present review aims to provide a summary of the current development of precision immunotherapy in sepsis. The review classifies sepsis patients into subgroups of prevailing pathways of pathophysiology and presents the clinical benefit described in recent RCTs where specific patient groups were treated with drugs tailored to their needs. Supporting evidence from the COVID-19 pandemic is also provided.

## Review

### Literature search

Cited references are from a literature search using the PubMed library filtered for the last 10 years. The main combinations of keywords used for the search were: sepsis/septic shock AND anakinra/TNF/IL-6/TREM-1 AND clinical trials; immunoparalysis AND sepsis/septic shock AND interferon-gamma/PD-1/anti-PD-1/IL-7 AND clinical trials; sepsis/septic shock AND vascular endothelium AND clinical trials; and sepsis AND extracorporeal removal AND clinical trials, where IL-1 refers to interleukin 1, TREM-1 is triggering receptor expressed on myeloid cells-1 and PD-1 is programmed death protein 1. Retrieved abstracts were reviewed by two of the co-authors (AA and DP) and the final references were selected to build this review.

Searches using the above terms retrieved the following: sepsis/septic shock AND anakinra AND clinical trials 18 results; sepsis/septic shock AND TNF AND clinical trials 141 results; sepsis/septic shock AND IL-6 AND clinical trials 248 results; sepsis/septic shock AND TREM-1 AND clinical trials 7 results; sepsis/septic shock AND interferon-gamma AND clinical trials 57 results; sepsis/septic shock AND PD-1 AND clinical trials 141 results; sepsis/septic shock AND anti-PD-1 AND clinical trials 10 results; sepsis/septic shock AND IL-7 AND clinical trials 15 results; sepsis/septic shock AND vascular endothelium AND clinical trials 37 results; and sepsis/septic shock AND extracorporeal removal AND clinical trials 27 results. The abstracts of the retrieved publications were reviewed and only those describing results of RCTs of interventions guided by specific biomarkers were retained.

### Pillars of sepsis immunotherapy

**Figure 1:**
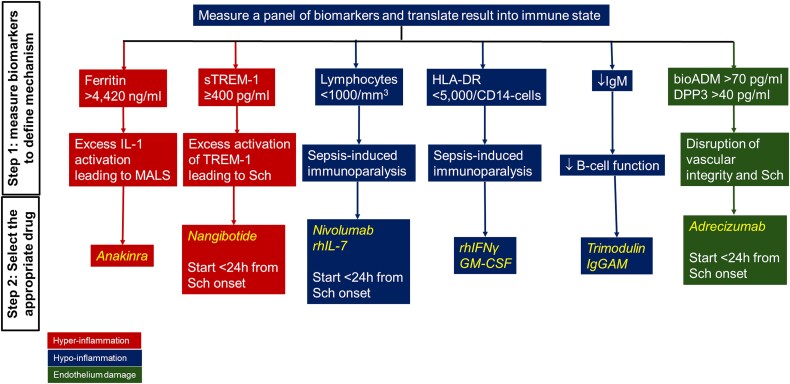
Rationale of precision immunotherapy in sepsis. The selection of precision immunotherapy has two steps. At the first step, every patient is subject to measurement of a wide panel of biomarkers. Based on the results, the patient is classified to a certain prevailing mechanism. At the second step, the most appropriate drug is selected based on mechanism classification. ↓: Decrease, *ADM* adrenomedullin, *DPP3* dimethyl-peptidase-3, *GM-CSF* granulocyte macrophage colony stimulating factor, *HLA* human leukocyte antigen, *IgGAM* IgM enriched immunoglobulin preparations, *IgM* immunoglobulin M, *IL* interleukin, *MALS* macrophage-activation-like syndrome, *rhIFNγ* recombinant human interferon-gamma, *rhIL-7* recombinant human IL-7, *Sch* septic shock, *sTREM-1* soluble triggering receptor expressed on myeloid cells-1

In the opinion of the authors, the main pillars of sepsis immunotherapy are as follows (Figure 1).

- The selection of the most appropriate patient using biomarkers. This means that information coming from biomarkers should be translated into the precise level of dysregulation of some specific pathway of the immune cascade.
- The selection of the best candidate drug that is able to restore the dysregulated pathway to normal function. The dose of the selected drug also needs to be considered.
- The appropriate time for administration of treatment.

#### The selection tool: endotypes or phenotypes

The traditional concept of sepsis pathogenesis considers that sepsis usually starts as an overwhelming hyper-inflammatory reaction of the host after microbial challenge that attenuates over time and leads to immunoparalysis [5]. This approach has been challenged over the years. Nowadays, we believe that when septic shock emerges, patients may fall into one of two extremes of immune activation: patients purely suffering from pro-inflammatory sepsis, patients purely suffering from anti-inflammatory sepsis or sepsis-induced immunoparalysis (SII) and patients who lie between these two extremes [6]. Patients with purely pro-inflammatory sepsis may present with macrophage activation-like syndrome (MALS) or the interferon-gamma (IFNγ)-driven sepsis endotype (IDS). The prevalence of MALS ranges between 5 and 10%. It is characterized by early progression to death; overall 28-day mortality is 65–80%. The classification of MALS is done by the hemophagocytosis score (HScore), developed for patients with macrophage activation syndrome secondary to malignancies, viral infections and rheumatologic disorders [7, 8]. MALS is driven by the excess production of interleukin (IL)-1 by tissue macrophages. Ferritin is the best classifier and blood levels >4420 ng/ml are diagnostic. This ferritin cut-off provides almost 100% specificity and 100% negative predictive value for classification, but very low sensitivity. This means that ferritin >4420 ng/ml provides certainty for inclusion of patients into trials for the management of sepsis-associated inflammation, but it cannot advise if patients with ferritin between 500 and 4420 ng/ml do not present with traits of hyper-inflammation driven by macrophage activation [7–9]. Several patients with MALS present with reciprocal negative feedback down-regulation of human leukocyte antigen (HLA)-DR on blood CD14-monocytes [10].

IDS is a pro-inflammatory endotype driven by the excess production of IFNγ which stimulates the production of the chemokine CXCL9 by tissue macrophages. CXCL9 is a secondary effector molecule associated with cell cytotoxicity. IDS is present in almost 20% of patients; it is characterized by the absence of down-regulation of HLA-DR on blood CD14-monocytes, ferritin does not reach excess levels and 28-day mortality is 40–43% [6].

SII is present in 30–40% of patients. The absolute number of HLA-DR receptors on CD14-moncytes is <5000/cell and 28-day mortality reaches up to 60%. The diagnosis of SII is based on the absolute number of HLA-DR receptors on the cell membrane of blood monocytes [11]. Quantibrite is an assay developed by BD using flow cytometry and holds one CE-IVD mark for the diagnosis of immunoparalysis [12]. Diagnosis is made when the number of HLA-DR receptors on CD45/CD14-monocytes is <8000; however, many investigators suggest that this cut-off should probably be lowered to 5000 for the accurate classification of SII [10].

There are many patients who are between the pro-inflammatory and anti-inflammatory extremes of this classification. These patients seem to have better prognosis. Most of these patients have mixed traits of pro-inflammation and anti-inflammation, like increased blood CXCL9 and decreased HLA-DR expression on CD14-monocytes or increased IFNγ without increased CXCL9 [6] (Table 1).

**Table 1:** Endotype classification of sepsis

**Endotype**	**Types of infections**	**Biomarker**	**Mechanism and clinical importance**	**Prevalence**	**28-day mortality**	**Ref.**
MALS	sCAP, BSI, HAP, IAI, VAP	Ferritin >4420 ng/ml	Excess production of IL-1 leading to early death	4–6% of all cases, 20% in Sch	65% in all cases; 80% in Sch	[6, 7, 10]
IDS	sCAP, BSI, HAP, IAI, VAP	IFNγ >3 pg/ml and CXCL9 > 2200 pg/ml	Excess production of IFNγ leading to the production of CXCL9	20% of all cases	40–43%	[6]
SII	sCAP, BSI, HAP, IAI, VAP	HLA-DR < 5000/CD14-monocytes and ferritin ≤ 4420 ng/ml	↓ Antigen presentation, apoptosis of lymphocytes, predisposing to secondary infections	25% of all cases, 43% of Sch	30% in all cases, 60% in Sch	[6, 10, 11]
SRS1	sCAP	*DYRK2*, *CCNB1IP1*, *TDRD9*, *ZAP70*, *ARL14EP*, *MDC1*, and *ADGRE3*	↓ HLA-DR genes; ↓ genes implicated in T-cell activation	35–41%	17–65%	[13]
SRS2	sCAP	*LAX1*, *TRIM44*, and *DDX24*		59–65%	17–41%	[13]
MARS1	sCAP	*BPGM:TAP2*	↓ Antigen presentation and T-cell signaling and death receptor signaling 37–44% with septic shock	29%	32–39%	[14]
MARS2	sCAP	*GADD45A:PCGF5*	↑ B-cell signaling 41–44% with septic shock	34–37%	22%	[14]
MARS3	sCAP	*AHNAK:PDCD10*	↑ PRR signaling 10–17% with septic shock	23–27%	14–23%	[14]
MARS4	sCAP	*IFIT5:GLTSCR2*	↑ IFN signaling 33% with septic shock	9–13%	5–33%	[14]

↓ Down-regulation, ↑ up-regulation, *BSI* bloodstream infection, *HAP* hospital-acquired pneumonia, *HLA* human leukocyte antigen, *IAI* intrabdominal infection, *IDS* interferon γ-driven sepsis, *IFN* interferon, *MALS* macrophage activation-like sepsis, *PRR* pattern recognition receptors, *sCAP* severe community-acquired pneumonia, *SII* sepsis-induced immunoparalysis, *VAP* ventilator-associated pneumonia

Ideally, all patients should be genomically analyzed for their endotype. Three systems of endotype classification have been suggested. The first system was introduced by Davenport *et al*. and classified patients with sepsis developing in the field of community-acquired pneumonia into sepsis response signatures 1 and 2. Sepsis response signature 1 resembles SII and has greater mortality [13]. Scicluna *et al*. classified patients into four endotypes MARS1 to MARS4 and showed that the MARS1 endotype is associated with worse outcome [14]. Sweeney *et al*. classified patients into the inflammopathic, adaptive and coagulopathic endotypes, characterized by hyper-inflammation, lymphocyte activation and excess coagulation respectively [15]. The limitations of endotype classification are that endotype analysis takes too much time, considering the need to act fast, it is expensive and it requires a high-level of training of laboratory staff. On the other hand, a protein biomarker can be analyzed by non-expert staff, provides fast results to guide the decision to treat and is much cheaper. In addition, it is not known whether a patient’s specific endotype remains stable over the course of the disease. If it does not, then treatment administration at a wrong time when the endotype has ceased to prevail may lead to treatment outcomes opposite to those expected. Patients with severe pneumonia by SARS-CoV-2 do not maintain a stable inflammopathic, adaptive or coagulopathic endotype, but shift over the first 7 days of the disease from one endotype to another [16].

Traditional medicine delivers treatment based on specific clinical features. A constellation of features can form a phenotype. Seymour *et al*. using four large-scale patient cohorts and applying a tool of 26 variables, classified sepsis into phenotypes α (alpha), β (beta) γ (gamma) and δ (delta). Patients of the δ phenotype have the worst outcome [17]. Using the same phenotypes, this classification was reproduced for patients with critical COVID-19 [18]. However, the exact biological pathway prevailing in each of the phenotypes remains to be defined.

#### Candidate drugs

The selection criteria for the best candidate drug vary considerably between investigators and criteria are often based on the mechanism that seems to prevail. One example is the ImmunoSep trial delivering immunotherapy tailored to the needs of each patient. Ferritin >4420 ng/ml is the classifier for MALS and guides treatment with anakinra. Anakinra is selected since it blocks IL-1 which is over-produced in MALS. HLA-DR <5000 receptors on CD45/CD14-monocytes is the classifier for SII and guides treatment with recombinant human (rh) IFNγ. rhIFNγ is selected since it may boost innate immune responses [19]. SII affects not only innate immune responses but also adaptive immune responses including processes like lymphocyte apoptosis, T-cell exhaustion and immunoglobulin production. This means that several other drug options may be available like growth factors, checkpoint inhibitors, recombinant IL-7 and intravenous immunoglobulin preparations enriched with immunoglobulin M (IgM) [20].

#### Appropriate time for administration of treatment

Choosing the appropriate time for administration of treatment is one of the most challenging aspects of immunotherapy. Although there is no real evidence on the appropriate time window, there is common belief that adjunctive immunotherapy in sepsis should start as fast as possible. One challenge to this belief comes from a subgroup analysis of the SAVE-MORE RCT in patients with severe pneumonia by SARS-CoV-2. In this RCT, patients are screened by blood levels of soluble urokinase plasminogen activator receptor which is a biomarker of excess activation of the IL-1 pathway. Patients with levels ≥6 ng/ml are randomized to blind treatment with placebo or anakinra adjunctive to SoC. Results showed that the primary endpoint (i.e. overall improvement of COVID-19 by day 28) was met irrespective of the quartile of time delay to the start of use of the study drug from the onset of symptoms [21]. This finding argues that as long as the selection biomarker remains increased, indicating that the pathogenesis pathway also remains active, the drug may also be active. However, in most RCTs treatment starts within the first 24 h from onset of sepsis or septic shock.

### Biomarker-driven immunotherapy: summary of the evidence

Evidence coming from RCTs over the last 10 years suggests that immune interventions in sepsis should be split into strategies aiming to attenuate exaggerated immune responses, restore SII and restore vascular tone.

#### Strategies aiming to attenuate exaggerated immune responses

In this strategy a biomarker is selected to identify patients who experience excess activation of one specific immune pathway. Then drugs blocking that pathway are administered (Table 2).

**Table 2:** Synopsis of the published efficacy of drugs aiming to attenuate the exaggerated host responses in sepsis guided by biomarkers

**Intervention**	**Patient population**	**Design**	**Treatment groups and dose regimens (n)**	**Endpoints**	**Efficacy**	**Ref.**
Anakinra	Adults with septic shock BSIs and infections of lung and abdomen Ferritin > 4420 ng/ml	RCT	IV Placebo (n = 20) IV Anakinra (n = 14), 200 mg q8h for 7 days	28-day mortality Exploratory analysis survival and SOFA decrease by day 7	85.7% vs 95.3% 10% vs 42.9%	[10]
Nangibotide	Adults with septic shock Infections of lung, abdomen and urinary tract Start of treatment the first 24 h from shock onset	RCT	Placebo (n = 12) IV Nangibotide 0.3 mg/kg/h (n = 13) after loading dose up to 12 h after vasopressor withdrawal IV Nangibotide 1 mg/kg/h (n = 12) after loading dose up to 12 h after vasopressor withdrawal IV Nangibotide 3 mg/kg/h (n = 12) after loading dose up to 12 h after vasopressor withdrawal	Safety Difference in total SOFA score from baseline until day 5	Drug-related TEAEs 17% vs 0% vs17% vs 0% Mean SOFA change −1.25 vs −2.91 vs −2.03 vs −0.9	[28]
Nangibotide	Adults with septic shock Infections of lung, abdomen and urinary tract Start of treatment the first 24 h from shock onset	RCT	Placebo (n = 116) IV low-dose nangibotide (n = 118): loading dose and 0.3 mg/kg/h up to 24 h after vasopressor withdrawal IV high-dose nangibotide (n = 121): loading dose and 1 mg/kg/h up to 24 h after vasopressor withdrawal	Difference in total SOFA score from baseline until day 5 Analysis for total population and high sTREM-1 (≥ 400 pg/ml)	No difference between groups When sTREM-1 ≥ 532 pg/ml, mean decrease 2.3 in favor of high-dose (*p* = 0.018) Possibly drug-related SAEs: 2% vs 3% vs 2%	[29]
Vilobelimab	Adults with septic shock Infections of lung and abdomen Start less than 6 h from onset of organ dysfunction or 3 h from onset of septic shock.		Placebo (n = 24) Vilobelimab two IV 2 mg/kg doses (0 and 12 h) (n = 16) Vilomelimab IV two 4 mg/kg doses (0 and 24h) (n = 16) Vilomelimab IV three 4 mg/kg doses (0, 24 and 72 h) (n = 16)	Safety 28-day mortality ICU-free days until day 28 Ventilator-free days until day 28 Vasopressor-free days until day 28	No difference between groups 12.5% vs 37.5% vs 18.8% vs 12.5% 2.5 vs 5.0 vs 19.5 vs 12.5 days 24 vs 10 vs 26.5 vs 24.5 days 23 vs 20.5 vs 27 vs 23 days	[31]

*BSI* Blood stream infections, *IV* intravenous, *n* number of patients, *q8h* every 8 hours *RCT* randomized controlled trial, *SAE* serious adverse event, *SOFA* sequential organ failure assessment, *TEAE* treatment-emergent adverse event, *sTREM* soluble triggering receptor expressed on myeloid cells, *ICU* intensive care unit

##### Anakinra

Anakinra is the recombinant human antagonist of the IL-1 receptor. The drug binds to the IL-1 receptor and attenuates the pro-inflammatory responses coming from cell signaling by IL-1α and IL-1β. A *post hoc* analysis of one RCT that took place almost 30 years ago and that failed to prove survival benefit in the overall patient population, showed that patients with features of MALS, classified by the co-presence of hepatobiliary dysfunction and disseminated intravascular coagulation, had survival benefit when treated with anakinra [22]. MALS is induced by the excess production of IL-1. When ferritin was developed as the diagnostic biomarker of MALS, one small-scale phase 2a RCT was designed where patients with septic shock and ferritin >4420 ng/ml received intravenously adjunctive treatment with placebo (n = 20) or anakinra (n = 14). The administered intravenous regimen was 200 mg every 8 h for 7 days. The trial failed to reach the primary endpoint, i.e. 28-day mortality. However, at the end of the 7-day treatment, 42.9%% of anakinra-treated patients were alive with a decrease in baseline sequential organ failure assessment (SOFA) score compared to 10% of placebo-treated patients (*p* = 0.042) [10].

Biomarker-guided anakinra treatment is approved for the treatment of severe COVID-19 pneumonia by the European Medicines Agency and by the US Food and Drug Administration based on the results of the SAVE-MORE RCT. Anakinra treatment met the primary endpoint of improvement of the World Health Organization Clinical Progression Scale by day 28 with 0.36 odds ratio. This was also associated with a decrease in 28-day mortality [23]. Treatment is guided by the biomarker soluble urokinase plasminogen activator receptor which detects IL-1 activation [24]. Patients treated with placebo had considerable changes from one endotype to another during the first 7 days of follow-up. Anakinra treatment stabilized the patients to the adaptive endotype which is characterized by efficient lymphocyte responses (odds ratio 2.31; *p* = 0.005). Remarkably, patients who after the first 7 days of treatment remained classified with the coagulopathic endotype had lower risk for progression into acute respiratory distress syndrome and mechanical ventilation if treated with anakinra [hazard ratio-(HR) 0.46, *p* = 0.024] [16]. Anakinra is the only drug, so far, for which associations with endotype trajectories have been studied.

##### Attenuation of the TREM-1 pathway

TREM-1 is highly activated on the cell membranes of blood neutrophils and monocytes and of endothelial cells of patients with septic shock. TREM-1 activation leads to the intracellular accumulation of calcium ions with parallel activation of DAP12 and the subsequent production of TNFα and of IL-8. The exact ligand of TREM-1 is not known. However, animal studies suggest that upon TREM-1 activation, tissue macrophages secrete the extracellular cold RNA-binding protein which acts as a danger-associated molecular pattern and further stimulates TREM-1 in an auto-inflammatory loop [25]. Survival of mice with sepsis induced after cecal ligation and puncture (CLP) was prolonged when treated with a peptide that inhibits extracellular cold RNA-binding protein–TREM-1 interaction. Following inhibition of TREM-1 activation in experimental endotoxemia, circulating neutrophil extracellular traps, like cell-free DNA and the myeloperoxidase-DNA complexes, decreased [26]. This happens through a direct effect on neutrophils, leading to a decrease in the intracellular efflux of calcium and the subsequent release of intracellular neutrophil extracellular traps.

Upon activation on myeloid cells, TREM-1 is cleaved into a soluble molecule, known as soluble (s)TREM-1. Blood concentrations of sTREM-1 are inversely correlated with the expression of HLA-DR on circulating CD14-monocytes, so that the higher the sTREM-1 level the greater the degree of SII. A prospective study in 116 patients with septic shock showed that sTREM-1 > 392 pg/ml 6–8 days after shock onset provided similar information to an absolute number of HLA-DR receptors < 6688 per CD14-monocyte, and is associated with greater risk of secondary infections (HR 3.61) [27].

Nangibotide is a 12-amino acid peptide that binds and blocks the biological activity of TREM-1. Safety of administration in septic shock was evaluated in a phase 2a study where the incidence of drug-related serious treatment-emergent adverse events was similar to that in patients treated with placebo. The change of SOFA score by day 5 of administration was numerically greater, albeit not significant, among patients treated with doses of 0.3 and 1 mg/kg/h [28]. The decrease in SOFA score following nangibotide treatment was greater among patients with increased sTREM-1 levels. These results encouraged a larger phase 2b study in septic shock where the change in SOFA score by day 5 was the primary endpoint and sTREM-1 blood levels were also measured at baseline. The primary endpoint was not met but a *post hoc* analysis revealed that the decrease in total SOFA score by day 5 was greater among patients treated with 1 mg/kg/h nangibotide with baseline blood sTREM-1 concentrations ≥ 532 pg/ml [29].

##### C5a inhibition

Although not guided by a specific biomarker, inhibition of the complement split product C5a by vilobelimab, a monoclonal antibody that blocks and inhibits the biological activity of C5a, appears promising. The drug was studied at different doses in small numbers of patients in a phase IIa RCT to assess pharmacokinetics and efficacy. Interestingly vilobelimab treatment decreased, albeit not significantly, the number of intensive-care-free days [30]. The drug was administered in the first 6 h from the onset of organ dysfunction.

Vilobelimab is approved by the US Food and Drug Administration for critical COVID-19. The drug is administered as six 800 mg intravenous infusions starting <48 h from mechanical ventilation. The 28-day mortality was 42% in the placebo group and 32% in the vilobelimab group [31].

##### IL-6 receptor inhibition

The *ex vivo* treatment of peripheral blood mononuclear cells of patients with severe COVID-19 with the IL-6 receptor inhibitor tocilizumab increases the expression of the HLA-DR receptor on CD14-monocytes and restores lymphopenia [32]. Tocilizumab has been approved for the management of critical COVID-19. Highest efficacy has been reported for patients with C-reactive protein between 75 and 150 mg/l. A recent meta-analysis of nine RCTs showed that tocilizumab decreases the relative odds for death by 11% (cumulative odds ratio 0.89); no toxicity was found [33].

##### Thymosin

Thymosin alpha1 inhibits Toll-like receptor-4 and has been administered together with ulinastatin in eight RCTs in patients with sepsis developing after urinary tract infections. A recent meta-analysis of these trials included in total 547 patients treated with a combination of ulinastatin and thymosin and 555 patients treated with placebo. Treatment decreased the odds for 28-day mortality (cumulative odds ratio 0.64) and for the duration of mechanical ventilation (cumulative odds ratio 0.58) [34].

A synopsis of the published efficacy and safety of drugs aiming to attenuate the exaggerated host responses in sepsis guided by biomarkers is provided in Table 2.

#### Strategies aiming to restore SII

The biomarkers of SII indicate specific features of immune exhaustion like down-regulation of antigen presentation and T-cell apoptosis. Administered drugs aim to reverse immune exhaustion (Table 3).

##### Nivolumab

The exhaustion of antigen-presenting cells, T-lymphocytes and B-lymphocytes are major components of SII. PD-1 negatively regulates the activation of lymphocytes and induces apoptosis. PD-1 exerts its function by binding to the receptor programmed death ligand-1 (PD-L1) and PD-L2. PD-L1 is widely expressed on different cells of the human body whereas PD-L2 is mainly expressed on antigen-presenting cells. Several prospective studies using flow cytometry have been done to study the expression of PD-1 and PD-L1/2 on immune cells. These studies compare the expression in patients with sepsis to healthy volunteers and also between sepsis survivors and sepsis non-survivors. Indeed, the expression of PD-1 is higher in CD4-lymphocytes and B-lymphocytes of patients compared with volunteers and also in the subset of memory B-lymphocytes expressing the CD27 receptor. Interestingly, PDL-1 and PDL-2 expression was not higher on B-lymphocytes of patients compared with volunteers [35]. The expression of PD-L1 is also pronounced on natural killer cells and on T-regulatory cells of non-survivors of sepsis compared with survivors [36, 37].

Nivolumab is a monoclonal antibody that blocks PD-1 and limits binding to its ligands. Two phase 1/2 studies have been done with the administration of single doses of 480 and 960 mg of nivolumab in patients with sepsis and low absolute lymphocyte counts. In both studies no drug toxicity was observed [38, 39] (Table 3). The selected time window for the start of nivolumab is at least 24 h from sepsis onset when the cessation of the pro-inflammatory host response is anticipated. The considerable cost of novilumab led to one pharmacokinetic simulation analysis which calculated that drug doses as low as 20 mg may effectively block PD-1 in sepsis [40].

**Table 3:** Synopsis of the published efficacy of drugs aiming to restore sepsis-induced immunoparalysis in sepsis guided by biomarkers

**Intervention**	**Patient population**	**Design**	**Treatment groups and dose regimens (n)**	**Endpoints**	**Efficacy**	**Ref**
Nivolumab	Sepsis ≥24 hours Hypotension requiring vasopressors ≥6 hours or ARDS requiring MV ≥24 hours or AKI Total lymphocytes <1100/mm^3^	RCT	IV single 480 mg nivolumab (n = 15) IV single 960 mg nivolumab (n = 16)	Safety Biomarkers	No safety concerns mHLA-DR >5000 mAB/cell by day 14 in all patients	[39]
Nivolumab	Sepsis ≥24 hours Hypotension requiring vasopressors ≥6 hours or ARDS requiring MV ≥24 hours or AKI Total lymphocytes <1100/mm^3^	RCT	IV single 480 mg nivolumab (n = 5) IV single 960 mg nivolumab (n = 8)	Safety Biomarkers	No safety concerns mHLA-DR >8000 mAB/cell by day 14 in all patients	[40]
CYT107	Septic shock requiring vasopressors SOFA ≥2 Total lymphocytes ≤900/mm^3^	RCT	Placebo (n = 10) IM CYT017 10 μg/kg once weekly (n = 8) for 4 weeks IM CYT017 10 μg/kg twice weekly (n = 9) for 4 weeks	Restoration of lymphopenia by week 4	Relative increase of total lymphocyte by 990 and 1300/mm^3^ Relative increase of CD4-lymphocyte by 320 and 280/mm^3^	[41]
Trimodulin	Community-acquired pneumonia Need for mechanical ventilation	RCT	Human albumin or placebo IV for 5 days Trimodulin (52 mg IgM/kg) IV for 5 days	Ventilator-free days 28-day mortality	8 vs 11 days 27.8% vs 22.2% *Post hoc* analyses for VFDs CRP ≥ 70 mg/l 9.2 vs 12.1 days (*p* = 0.044) IgM ≤ 0.8 g/l 8.9 vs 12.4 days (*p* = 0.031) CRP ≥ 70 mg/l + IgM ≤ 0.8 g/l 8.7 vs 12.6 days (*p* = 0.031) PCT ≥ 2 ng/ml 7.8 vs 11.2 days (*p* = 0.047) *Post hoc* analyses for 28-day mortality CRP ≥ 70 mg/l 30.5% vs 13.8% (*p* = 0.030) IgM ≤ 0.8 g/l 30.9% vs 14.3% (*p* = 0.043) CRP ≥ 70 mg/l + IgM ≤ 0.8 g/l 36.6% vs 11.8% (*p* = 0.006) PCT ≥ 2 ng/ml 31.4% vs 23.3% (*p* 0.489)	[42]
GM-CSF	Septic shock requiring vasopressors mHLA-DR <8000/CD14-monocytes	RCT	Placebo (n = 44) Sargramostim sc 125 μg/m^2^ daily for 5 days (n = 54)	Incidence of new ICU-acquired infections 28-day mortality Immune status	11% vs 11% 27% vs 24% Significant HLA-DR increase in the intervention group	[43]
GM-CSF	Decompensated cirrhosis Spontaneous bacterial peritonitis >250 neutrophils/mm^3^ of ascitic fluid		Meropenem (n = 66) Meropenem + IV sargramostim 1.5 mg/kg/day (n = 65)	<250 neutrophils/mm^3^ of ascitic fluid by 48 hours New-onset infections Resolution of co-existing pneumonia	31.8% vs 60% (*p* = 0.001) 37.9% vs 21.5% (*p* = 0.05) 10.5% vs 47.05% (*p* = 0.02)	[44]

*AKI* acute kidney injury, *ARDS* acute respiratory distress syndrome, *CRP* C-reactive protein, *GM-CSF* granulocyte monocyte colony stimulating factor, *ICU* Intensive Care Unit, *IgM* immunoglobulin M, *IM* intramuscular, *IV* intravenous, *mHLA-DR* expression of human leukocyte antigen-DR on monocytes, *MV* mechanical ventilation, *PCT* procalcitonin, *RCT* randomized controlled trial, *sc* subcutaneous, *SOFA* sequential organ failure assessment, *VFDs* ventilator-free days

##### Restoration of lymphocyte function

Failure of function of lymphocytes is characteristic of SII and may be expressed as either low counts of lymphocyte subsets or a decrease in immunoglobulins. IL-7 stimulates the proliferation of T- and B-lymphocytes. In the IRIS-7 RCT, CYT017, a recombinant form of IL-7, was administered at both low and high doses in patients with septic shock and lymphopenia. The trial met the primary endpoint of resolution of lymphopenia by the end of treatment [41].

Efficient phagocytosis of the invading pathogens requires opsonization, which often fails in sepsis due to decreases in circulating IgM and IgG. In the CIGMA RCT, patients with severe community-acquired pneumonia necessitating mechanical ventilation were randomized to treatment with adjunctive placebo or trimodulin. Trimodulin is a polyclonal antibody preparation enriched in IgM (23%). The study did not meet the primary endpoint of increase of ventilator-free days with trimodulin administration. However, in the subgroup of patients with IgM ≤ 0.8 g/l, 28-day mortality was decreased [42].

##### Growth factors

The idea of treatment with growth factors is based on the need to stimulate neutrophil phagocytosis and prevent secondary infections in patients with SSI. Two trials have reported results in patients with septic shock and in patients with difficult-to-treat (DTT) spontaneous bacterial peritonitis (SBP). Sargramostin was given for 5 days in patients with septic shock and low HLA-R expression on CD14-monocytes. Compared to placebo treatment, sargramostin did not reduce the incidence of secondary infections [43]. DTT SBP is defined as SBP presenting in patients with decompensating liver cirrhosis resulting from one community-acquired infection or from one hospital-acquired infection and which is unresponsive to commonly used antibiotics. In a recent RCT, patients with DTT SBP and decompensated liver cirrhosis were randomized to treatment with meropenem coupled to adjuvant placebo or granulocyte monocyte colony stimulating factor. The addition of granulocyte monocyte colony stimulating factor increased the rate of early clinical response [44] (Table 3).

The IL-4 fusion protein to apoprotein A1 (ApoA1-IL-4) seems a promising strategy to reverse SSI. IL-4 is an anti-inflammatory cytokine but addition to monocytes pre-treated with LPS managed to maintain adequate production of TNFα. Addition of IL-4 to the monocytes of a human subject to experimental endotoxemia managed to restore capacity for the production of TNFα. Pharmacokinetic studies in mice injected with APoA1-IL-4 showed accumulation in the kidney and liver [45].

A synopsis of the published efficacy and safety of drugs aiming to restore SII guided by biomarkers is provided in Table 3.

#### Strategies aiming to restore vascular tone and maintain endothelial permeability

Current evidence suggests that there are two bioactive molecules that impact vascular tone and vessel permeability. The first molecule is adrenomedullin (ADM) which enhances endothelial barrier function. When ADM crosses the vascular endothelium and moves to the interstitial space, it induces the relaxation of vascular smooth muscle cells and decreases arterial pressure. The second molecule is cytoplasmic dipeptidyl-peptidase 3 (DPP3) which rapidly hydrolyses molecules, like angiopoietin 1, that maintain vascular integrity at the intravascular compartment [46].

The large-scale multinational AdrenOSS-1 study showed that both levels of bioactive ADM (bioADM) and DPP3 are increased early in septic shock and that their blood levels predict an unfavorable outcome [47, 48]. More precisely, bioADM > 70 pg/ml and DPP3 > 40 pg/ml are risk factors for 28-day mortality. This risk classification is even validated when patients experience a drop of lactate levels to <2 mmol/l in the first 24 h. These patients are anticipated to improve when the bioactive ADM level is >70 pg/ml [49].

Two monoclonal antibodies that target DPP3 and bioADM seem to be promising in the treatment of septic shock. Procizumab blocks the function of DPP-3. When studied in an animal model of sepsis induced by CLP, survival was prolonged and this was associated with a decrease in myocardial oxidative stress [50]. Adrecizumab blocks ADM and does not allow ADM to cross into the interstitial space. In this way adrecizumab treatment keeps high levels of ADM at the intravascular space and maintains endothelial barrier integrity. Adrecizumab was given as treatment in experimental sepsis induced after endotoxemia and CLP. Adrecizumab treatment decreased the leakage of albumin from blood vessels in the liver and kidney, decreased tissue levels of vascular endothelial growth factor and increased tissue concentrations of angiopoietin 1 [51].

The AdrenoSS-2 RCT is an example of precision management of septic shock. Patients starting vasopressors at <12 h and with blood bioADM of > 70 pg/ml were randomized to treatment with single doses of placebo (n = 152), 2 mg/kg adrecizumab (n = 72) or 4 mg/kg adrecizumab (n = 77). Efficacy results of both doses of adrecizumab were not significant when analysis comprised the entire intent-to-treat population (HR 0.837). However, the HR for 28-day mortality was close to significance among the SOFA-adjusted population (HR 0.59) [52].

### Major future steps

Search at Clinicaltrials.gov retrieved three registrations of RCTs of precision immunotherapy in sepsis: ImmunoSep, titrated administration of IgM-enriched preparation and PALETTE. ImmunoSep is a double-blind, double-dummy RCT comparing the efficacy of precision immunotherapy for patients with sepsis developing in the field of lung infection or primary bacteremia. Study participants are randomized to treatment with SoC and precision immunotherapy or SoC and placebo immunotherapy. Both groups receive two interventions: one intravenous and another subcutaneous. Patients randomized to the precision immunotherapy arm receive intravenous anakinra and subcutaneous placebo if suffering from MALS, and intravenous placebo and subcutaneous rhIFNγ if suffering from SII. Patients randomized to the placebo arm receive both intravenous and subcutaneous placebo interventions. SoC is given according to the Surviving Sepsis Campaign guidelines [19]. The primary endpoint is an at least 1.4-point decrease of the mean SOFA score on day 9 compared to baseline SOFA score. The study has finished enrolment and results are expected soon.

In the IgM-fat trial, patients who meet the Sepsis-3 definitions for septic shock and with serum IgM < 60 mg/dl receive treatment within the first 24 h from the start of vasopressors with placebo or a standard dose of the IgM-enriched pentaglobin preparation or adjusted doses of pentaglobin, with the aim of maintaining serum IgM > 100 mg/dl [53]. The primary endpoint is all-cause 28-day mortality. This study has not started yet. Finally, PALETTE is a platform trial for patients with sepsis irrespective of etiology aiming to study the efficacy of several treatments (anakinra, corticosteroids and tocilizumab) for 28-day mortality [54]. PALETE has not started yet.

## Conclusions

The above analysis suggests that studying adjunctive treatments in sepsis requires a change of mentality. The design of RCTs includes two stages: at the screening stage biomarkers are measured and their levels dictate the most likely pathway of pathogenesis at the specific timepoint of screening; at the enrolment stage precision immunotherapy starts with the aim of modulating this pathway. These RCTs seem to represent a big hope towards maximizing treatment efficacy for specific patient populations, minimizing adverse events and maximizing cost benefit. Although no specific trials have been published in patients with burns or traumas, the analyzed results may be extrapolated to patients with sepsis developing after burns or trauma. The emerging enthusiasm for immunotherapy during the COVID-19 pandemic does not mean that the efficacy of drugs like anakinra, tocilizumab and dexamethasone can be extrapolated to bacterial sepsis. In many cases viral sepsis after SARS-CoV-2 infection does not necessarily meet the complexity of bacterial sepsis. Following infection by SARS-CoV-2 patients enter a pulmonary phase of rapid viral dissemination in the lung followed by hyper-inflammation and critical illness. On the other hand, bacterial sepsis is characterized by prevailing hyper-inflammation or anti-inflammation while several patients present with traits of both hyper- and hypo-inflammation.

## Abbreviations

ADM: Adrenomedullin; bioADM: Bioactive adrenomedullin; CLP: Cecal ligation and puncture; CRP: C-reactive protein; DPP: Dipeptidyl-peptidase 3; DTT: Difficult-to-treat; HR: Hazard ratio; IDS: Interferon-gamma-driven sepsis; IgM: Immunoglobulin M; IFNγ: Interferon γ; IL-1: Interleukin 1; MALS: Macrophage activation-like syndrome; PD-1: Programmed death protein 1; PD-L1: Programmed death ligand-1; RCT: Randomized controlled trial; SBP: Spontaneous bacterial peritonitis; SII: Sepsis-induced immunoparalysis; SOFA: Sequential organ failure assessment; sTREM-1: Soluble triggering receptor expressed on myeloid cells-1; TNF: Tumor necrosis factor; TREM-1: Triggering receptor expressed on myeloid cells-1.
